# Association of Indoleamine 2,3-Dioxygenase (IDO) Activity with Outcome after Cardiac Surgery in Adult Patients

**DOI:** 10.3390/metabo14060334

**Published:** 2024-06-14

**Authors:** Andrea Stieger, Markus Huber, Zhanru Yu, Benedikt M. Kessler, Roman Fischer, Lukas Andereggen, Beatrice Kobel, Frank Stueber, Markus M. Luedi, Mark G. Filipovic

**Affiliations:** 1 Department of Anaesthesiology and Pain Medicine, Cantonal Hospital of St. Gallen, 9007 St. Gallen, Switzerland; markus.luedi@extern.insel.ch; 2 Department of Anaesthesiology and Pain Medicine, Inselspital, Bern University Hospital, University of Bern, 3010 Bern, Switzerland; markus.huber@insel.ch (M.H.); beatrice.kobel@insel.ch (B.K.); frank.stueber@insel.ch (F.S.); mark.filipovic@insel.ch (M.G.F.); 3 Target Discovery Institute, Centre for Medicines Discovery, Nuffield Department of Medicine, University of Oxford, Oxford OX3 7FZ, UK; zhanru.yu@ndm.ox.ac.uk (Z.Y.); benedikt.kessler@ndm.ox.ac.uk (B.M.K.); roman.fischer@ndm.ox.ac.uk (R.F.); 4 Department of Neurosurgery, Cantonal Hospital of Aarau, 5000 Aarau, Switzerland; lukas.andereggen@gmail.com

**Keywords:** indoleamine 2,3-dioxygenase, cardiopulmonary bypass, cardiac surgery, tryptophan catabolism, outcome

## Abstract

Indoleamine 2,3-deoxygenase (IDO) plays an important role in the catabolism of the amino acid tryptophan. Tryptophan and its metabolites are key immune modulators. Increased IDO activity has been observed in various diseases and is associated with worse clinical outcomes. However, comprehensive research regarding its role in cardiac surgery remains limited. Therefore, we aimed to investigate perioperative changes in IDO activity and pathway metabolites, along with their impact on clinical outcomes in adult patients undergoing cardiac surgery. As an observational cohort study conducted at the Inselspital in Bern from January to December 2019, we retrospectively analyzed the data of prospectively collected biobank samples of patients undergoing cardiac surgery with the use of cardiopulmonary bypass. IDO pathway metabolite analysis was conducted by mass spectrometry. Perioperative dynamics were descriptively assessed and associated with pre-defined clinical outcome measures (30-day mortality, 1-year mortality, incidence of stroke and myocardial infarction, and length of hospital stay) through a multi-step exploratory regression analysis. A cohort of 192 adult patients undergoing cardiac surgery with the use of cardiopulmonary bypass were included (median age 67.0, IQR 60.0–73.0, 75.5% male). A significant perioperative decrease in the kynurenine/tryptophan (Kyn/Trp) ratio (−2.298, 95% CI −4.028 to −596, *p* = 0.009) and significant perioperative dynamics in the associated metabolites was observed. No association of perioperative changes in IDO activity and pathway metabolites with clinical outcomes was found. A significant decrease in the Kyn/Trp ratio among adult patients undergoing cardiac surgery indicates a perioperative downregulation of IDO, which stands in contrast to other pro-inflammatory conditions. Further studies are needed to investigate IDO in the setting of perioperative immunomodulation, which is a key driver of postoperative complications in cardiac surgery patients.

## 1. Introduction

Indoleamine-2,3-Dioxygenase (IDO) is an enzyme that plays a crucial role in the catabolism of the essential amino acid tryptophan. Tryptophan is required for the synthesis of serotonin and serves as an immune booster. The main function of IDO is to break down tryptophan (TRP) in N-formylkynurenine (KN), which is further metabolized into kynurenic acid (KA), quinolinic acid (QA), and picolinic acid (PA) [1,2]. These metabolites exhibit immunosuppressive qualities by inhibiting T-cell proliferation and thereby preventing excessive inflammation and tissue damage [1]. Inflammation itself leads to an activation of the kynurenine pathway and an increase in IDO expression [3,4]. To measure IDO activation, the serum kynurenine to tryptophan (Kyn/Trp) ratio is principally used instead of the direct measurement of IDO activity, which is more complex to achieve [5].

The role of IDO has been extensively investigated in various diseases, including cancer [3,6,7,8,9,10,11,12,13,14,15], autoimmune disorders [16,17], and neurodegenerative conditions [18,19]. In cancer, elevated IDO levels correlate with a spread to the lymph nodes [20], increased tissue invasion, and metastasis formation [3,11,21]. During inflammatory reactions, such as in influenza-induced lung inflammation [22] or in sepsis, IDO activity is increased and is associated with higher mortality rates [23]. The surgical trauma-induced stress response leads to endocrine changes and tissue damage, resulting in an inflammatory response [24].

Cardiac surgery with the use of cardiopulmonary bypass (CBP) is another externally induced condition which triggers massive systemic inflammation by activating inflammatory leukocytes due to high shear stress, potentially leading to a prolonged hospital stay and multiorgan dysfunction [25]. Moreover, there is evidence that CPB results in postoperative immunosuppression due to induced lymphocyte dysfunction, leading to an increased susceptibility to infections [26]. This immune response is fueled by multiple factors such as the surgical trauma itself, blood interaction with extracorporeal circuits, and reperfusion injury [27], and has repeatedly been shown to influence postoperative outcomes [27,28,29]. IDO’s involvement in inflammatory responses and in conditions associated with heightened immune activation, such as CPB surgery, might serve as an immune marker and potential therapeutic target [3,4,30,31,32].

However, despite its potential, research investigating IDO in the context of cardiac surgery remains scarce. A study by Lesouhaitier et al., investigating CPB-induced immunosuppression, observed elevated IDO activity after CPB in adult patients, although inhibiting IDO did not restore the ability of T-cells to proliferate ex vivo [26]. Another study involving a pediatric cohort undergoing CPB detected slightly elevated IDO activity perioperatively compared to healthy controls undergoing non-cardiac surgery [33]. Therefore, the role of IDO in the immune response to cardiac surgery, especially in adult patients, remains largely unknown. Additionally, the complexity of inflammatory mechanisms involving various metabolites, interactions, and inter-related pathways poses challenges for the characterization, detection, and description of comprehensive immune mechanisms. Hence, previous studies have focused on limited sets of tryptophan pathway metabolites. In this context, mass spectrometry has recently been used to describe immunological stress after cardiac surgery, as it includes the ideal means to characterize complex conditions such as inflammatory responses to CPB [34]. Moreover, it allows the association of potential dynamics in perioperative metabolites with postoperative outcomes in a highly innovative approach.

Based on these findings, the aim of this report was to investigate the perioperative dynamics of tryptophan pathway metabolites in adult patients undergoing cardiac surgery with the use of cardiopulmonary bypass using an innovative mass spectrometry methodology. Moreover, we aimed to characterize IDO activity with the help of the Kyn/Trp ratio in the perioperative phase. As a clinical outcome, we wanted to associate IDO activity and individual metabolites with clinical outcome measures in an exploratory analysis.

## 2. Material and Methods

### 2.1. Study Population

This study is a retrospective analysis of a prospective observational cohort study from the Bern Perioperative Biobank (ClinicalTrials.gov; NCT04767685), including 192 patients who underwent cardiopulmonary bypass surgery at the Bern University Hospital between January and December 2019. The study was approved by the local ethics committee (Cantonal Ethics Commission of Bern, Bern, CH-KEK Nr. 2018-01272 for sampling and KEK Nr. 2019-2000 for data analysis), and informed consent was provided prior to study inclusion. The cohort has been extensively described previously [34,35,36]. Inclusion criteria were elective cardiac surgery and the provision of written informed consent. Cardiac surgery included surgery of the ascending aorta or aortic arch as well as coronary artery bypass grafting (CABG) and repair of the mitral (MVR), aortic (AVR), and tricuspid (TVR) valves. All patients received a median sternotomy and cardiopulmonary bypass with conventional extracorporeal circulation circuits (CECCs) or minimally invasive extracorporeal circulation circuits (MIECCs). Exclusion criteria were emergency surgery and confirmed or suspected pregnancy.

With regard to sample size, the current cohort represents a convenience sample of prospectively enrolled patients. No formal sample size was considered—the associated limitations are discussed further below.

Reporting adhered to the Strengthening the Reporting of Observational Studies in Epidemiology (STROBE) Statement [37].

### 2.2. Blood Sampling

Blood samples were obtained before the induction of general anesthesia (preoperative), upon discontinuation of cardiopulmonary bypass (intraoperative), and 24 h post-surgery (postoperative). The samples were subsequently stored at the Bern Liquid Bank at −80 °C.

### 2.3. Mass Spectrometry Analysis

Chemicals and metabolic standards

2-Propanol was purchased from Fisher Chemical (Loughborough, UK). Trifluoroacetic acid (TFA) was purchased from Sigma-Aldrich (Darmstadt, Germany). N-Methyl-N-(trimethylsilyl)trifluoroacetamide was purchased from FluoroChem Ltd. (Hadfield, UK). Chlorotrimethylsilane was purchased from Merck (Darmstadt, Germany). The metabolic standards used in this study were purchased from Sigma Aldrich (Darmstadt, Germany) with the exception of the following: sodium kynurenate (Abcam PLC, Cambridge, UK) and myristic acid-14,14,14-d_3_ used as an internal standard (Cambridge isotope Laboratories, Tewksbury, MA, USA).

Sample preparation

Plasma (100 μL) was added to 2-propanol (800 μL) with myristic acid-d_3_ (1 μg/mL) and trifluoroacetic acid (TFA, 0.5% *v*/*v*). The mixtures were vortexed using a bead beater (Precellys 24, Bertin Technologies, Paris, France) for three cycles (5000 Hz, 20 s) with a dry ice bath between the cycles. All suspension material was kept at −80 °C overnight. The mixtures were centrifuged for 30 min at 16,000× *g* at 4 °C. The supernatant was collected and added to a glass vial and dried under a vacuum (GeneVac EZ-2 Evaporator) for 4 h. The dried samples were kept at −80 °C until further processing. The samples were desiccated in a SpeedVac for 1 h prior to chemical derivatization.

Chemical derivatization

The samples were resuspended in a mixture of 40 μL N-Methyl-N-(trimethylsilyl)trifluoroacetamide with 5% chlorotrimethylsilane and 40 μL pyridine, followed by incubation for one hour at 60 °C with shaking at 1200 rpm. The samples were cooled to ambient temperature and centrifugated at 8000× *g* for 30 min at 4 °C. The samples were immediately used for GCxGC-MS analysis.

GCxGC-MS analysis

The samples were analyzed using a two-dimensional gas chromatography single-quad mass spectrometer (GCxGC-MS) system as described previously [38]. In brief, the GCxGC-MS system comprised a gas chromatograph coupled to a quadrupole mass spectrometer GCMS QP2010 Ultra (Shimadzu, Milton Keynes, UK) and equpiied with an AOC-20i/s auto sampler (Shimadzu, Milton Keynes, UK). The first-dimension separation was carried out on a SHM5MS capillary column (30 m × 0.25 mm i.d. × 0.25 μm film thickness, Shimadzu), while the second-dimension separation was performed on a BPX-50 capillary column (5 m × 0.15 mm i.d. × 0.15 μm film thickness, SGE). Helium was used as a carrier gas at a 73 psi constant inlet head pressure. The modulation period was set as 6 s. The samples (1 μL) were injected at 280 °C. The oven temperature was programmed as follows: (1) starting at 100 °C held for 2 min, from 100 to 220 °C at 30 °C/min, held at 220 °C for 1 min, then to 280 °C at 5 °C/min, held at 280 °C for 1 min, then to 300 °C at 40 °C/min, and held at 300 °C for 2 min; and (2) from 60 °C to 320 °C at 10 °C/min unless stated otherwise and held at 320 °C for 8 min. The interface temperature of the mass spectrometer was set at 300 °C, and the ion source was heated at 230 °C. The MS was operated at a scan speed of 20,000 amu covering a range of *m*/*z* 50–520. Electron Ionization spectra were recorded at 70 eV.

Data processing and analysis

Raw GCxGC MS data were processed using GCMSsolution software (v2.72/4.20 Shimadzu) and ChromSquare software (v2.1.6, Shimadzu) in combination with the NIST 11/s, OA_TMS, FA_ME, and YUTDI in-house libraries that were used for data analysis as described previously [38]. The annotation of metabolites was carried out by comparing them to external standards (IM spectra and retention times adjusted to the internal standard myristic acid-14,14,14-d_3_) and by spectrum-matching-based searches with the above databases for those metabolites without external standards. The similarity score threshold was set to 80 (out of 100), and the confidence of identification was further validated by the manual inspection of matches between the experimentally observed and reference EI spectra. The fragment ions for the quantitation of the metabolites are shown in Table 1 [38].

### 2.4. Other Study Variables

All relevant preoperative, perioperative, and postoperative data for each patient were collected from electronic patient charts (Dendrite Clinical Systems Ltd., Henley on-Thames, UK).

### 2.5. Clinical Outcome Measures

Clinical outcome measures were defined as all-cause 30-day and 1-year mortality, the incidences of myocardial infarction and stroke, and the length of hospital stay. Myocardial infarction was assessed in accordance with the fourth universal definition of myocardial infarction [39]. Stroke was defined as an acute clinically apparent neurological deficit accompanied by imaging confirmation of bleeding or cerebral ischemia. The incidences of myocardial infarction and stroke were followed until hospital discharge.

### 2.6. Statistical Analysis

In terms of summary measures, the perioperative characteristics of the kynurenine/tryptophan ratio and IDO pathway metabolites were represented with median values and associated 95% confidence intervals of the medians for each time point.

The overall magnitude of the perioperative changes was assessed with Kendall’s W for the effect size and a *p*-value based on the Friedman Test, thus accounting both for non-normally distributed values and the repeated-measures design of this study. Pairwise contrasts and associated 95%-confidence intervals with respect to the three time points were calculated with the Wilcoxon signed-rank test.

The associations of the kynurenine/tryptophan ratio and IDO pathway metabolites with clinical outcome measures were based on a multivariable regression analysis using either logistic regression (in the case of binary outcomes) and linear regression (in the case of length of stay). For the length of stay, the log-transformed duration was used in the regression analysis due to the observed skewness in the distribution. We adjusted for age, sex, and body mass index (BMI) in the multivariable regression models. For our exploratory analysis, we assessed the adjusted associations with respect to other comorbidities, medication use, and surgery-related covariates (ECC vs. MiECC, betablockers, bypass time, and aortic valve) by including them one by one in the pre-defined set of confounders (age, sex, and BMI). These analyses are provided in the Supplementary Material (Tables S1–S4).

Given the exploratory nature of our associations, no *p*-value adjustments for multiple comparisons were implemented. The statistical analyses were performed with R version 4.0.2.

## 3. Results

### 3.1. Study Population

A total of 192 patients were included. The median age was 67.0 years (interquartile range 60.0, 73.0) and most patients were male (75.5%). The baseline characteristics of this population have been described previously [34,35,36]. Details are presented in Table 2.

### 3.2. Perioperative Dynamics of Kynurenine/Tryptophan Ratio and IDO Pathway Metabolites

**Figure 1:**
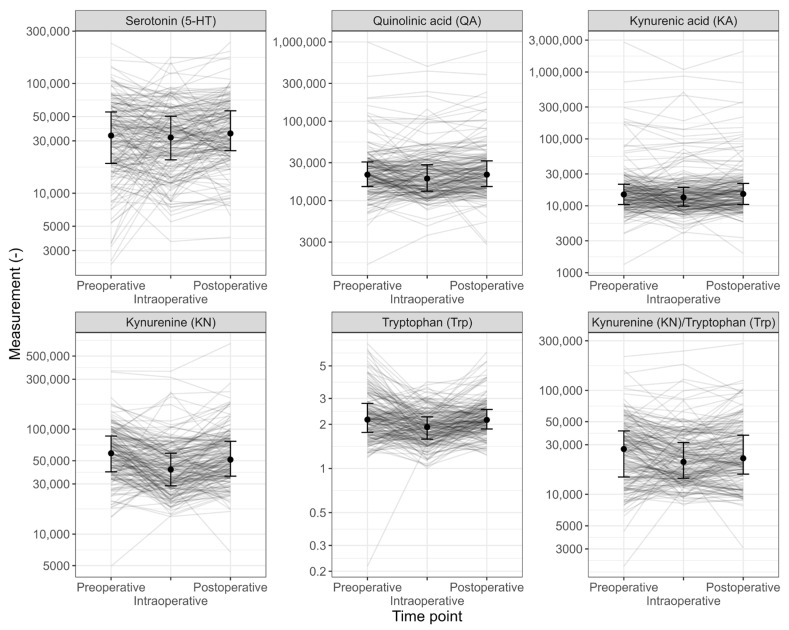
Graphical summary measures of the Kyn/Trp ratio and the 5 metabolites, and an assessment of the perioperative changes.

Significant perioperative dynamics in all tryptophan pathway metabolites were observed. Details are shown in Table 3 and Figure 1. The Kyn/Trp ratio showed a significant decrease with a preoperative level of 27.282 (95% CI 23.553 to 30.667) and a postoperative level of 22.211 (95% CI 20.166 to 25.400), resulting in an effect size of 0.07 (*p* < 0.001). In the paired analysis, this translated to a perioperative decline in the Kyn/Trp ratio of −2.298 (95% CI −4.028 to −596, *p* = 0.009) from the postoperative to preoperative levels. Specific analyses for patients with coronary artery disease did not reveal differences in IDO activity, or kynurenine levels, compared to patients without coronary artery disease (Supplementary Materials Figures S1 and S2).

### 3.3. Association of Kynurenine/Tryptophan Ratio and IDO Pathway Metabolites with Clinical Outcome Measures

An association between the levels of the individual metabolites or the Kyn/Trp ratio and the clinical outcome measures was not found (Table 4).

## 4. Discussion

We show that tryptophan metabolism is strongly influenced by cardiac surgery with the use of cardiopulmonary bypass in adults. All measured metabolites exhibited significant perioperative changes in their levels. In addition, a significant decrease in the Kyn/Trp ratio was found, suggesting a downregulation of IDO activity during the perioperative period.

These findings stand in contrast to other pro-inflammatory states, which are associated with an increase in IDO activity. In cases of severe sepsis, characterized by prolonged immune dysregulation, the activity of IDO gradually increases in proportion to the severity of sepsis [23]. Furthermore, IDO is highly expressed in patients with various cancers [3]. For example, individuals with colorectal cancer exhibit lower tryptophan levels and a higher level of the Kyn/Trp ratio [21]. Patients with glioma demonstrate elevated IDO expression, and a higher level of IDO predicts a worse outcome, particularly in terms of survival [40]. Consequently, the activation of the kynurenine pathway is associated with a poorer prognosis in cancers, correlating with tumor angiogenesis, invasion, and metastasis formation [3].

Surgical trauma induces an acute stress reaction in the body similar to that seen in an acute infection. The damage to tissue triggers the activation of various immune cells and cytokines [24]. Similarly, CBP in cardiac surgery is another driver of both inflammatory and immunosuppressive mechanisms: the exposure of blood to the surface of the CPB circuit leads to direct-contact activation of the immune system. Aortic cross-clamping and, in particular, its release is associated with the activation of cytokines in the inflammatory response. Furthermore, splanchnic hypoperfusion during CPB may result in the gut translocation of endotoxin, leading to a stimulation of the inflammatory response [41,42]. This process leads to a direct activation and release of many inflammatory mediators including oxygen-free radicals, arachidonic acid metabolites, cytokines, platelet-activating factor, nitric oxide, and endothelin [43]. Patients undergoing cardiac surgery with extracorporeal circuits also have a higher risk of infection, SIRS, and sepsis underlining the postoperative immunosuppressive phase [44]. As an example, a study conducted by Nguyen et al. indicated that higher preoperative neutrophil counts and low perioperative lymphocyte counts were associated with the occurrence of postoperative complications in patients undergoing cardiac surgery [45]. Therefore, one might hypothesize that IDO activity would also increase during cardiac surgery with the use of cardiopulmonary bypass. However, this was not the case in our population.

Comparative data to other studies investigating IDO activity in cardiac surgery patients remain scarce. A study investigating immune suppression following CPB in 43 adult patients found higher IDO levels postoperatively than preoperatively, indicating a T-cell dysfunction; however, the addition of an IDO inhibitor ex vivo showed no benefit [26]. Sabapathy et al. conducted a study in 2021 involving 51 infants aged 31 days to 2 years undergoing CPB surgery for single-ventricle heart disease (SVHD). They reported a higher Kyn/Trp ratio in this group compared to healthy controls undergoing non-cardiac surgery, along with elevated levels of proximal kynurenine pathway metabolites [33]. However, IDO was already upregulated at baseline in this cohort. Children with SVHD tend to have a distinct metabolomic fingerprint, including tryptophan metabolism, compared to other children [46]. Therefore, comparing these findings to our study may be challenging due to significant differences between the investigated pediatric population and adult patients.

While those studies have focused on investigating the Kyn/Trp ratio or only selected metabolites, we have comprehensively assessed an array of downstream metabolites in the pathway. Our findings that the change in ratio is mainly driven by a reduction in kynurenine while the perioperative levels of tryptophan remain stable support the theory of decreased tryptophan metabolism during CBP. Together with a trend of higher levels of downstream metabolites, the increased metabolism of kynurenine could also be responsible for the dynamics in the Kyn/Trp ratio. Consequently, the role of IDO in the context of cardiac surgery may differ from its role in other pro-inflammatory conditions such as cancer. As a possible explanation, it was shown that IDO is induced mainly through Interferon γ [5], while cardiac surgery suppresses the expression of Interferon γ [47]. In this context, the term T helper 1 (TH1)/TH2 cytokine expression shift describes the reduced activity of Interferon γ TH1 cells and the increased activity of TH2 cells, which have been shown in patients after major surgical procedures such as on-pump cardiac surgery [48,49]. Furthermore, the shifts towards TH2 cells and T-cell imbalance have been associated with post-traumatic sepsis [50]. While T-cell impairment due to the inhibition of T-cell proliferation has been advocated as a major factor responsible for immunosuppression and even death in patients with sepsis [51], there is evidence that in cardiac surgery, the hyporesponsiveness may be explained rather as a result of altered cell function than as a result of absolute cell count changes [48]. Therefore, perioperative dynamic changes in the number and ratio of T lymphocytes may provide a potential explanation for the alterations in IDO activity observed in this cohort, linking cardiac surgery with postoperative immunosuppression and differentiating it from other pro-inflammatory conditions.

In view of these findings, we can infer that IDO plays an important role in the complex interplay of postoperative immunomodulation after cardiac surgery. This is interesting, as IDO can be selectively targeted and inhibited [3,4,30,31,32], and therefore a therapeutic intervention might potentially mitigate postoperative complications. Nevertheless, we were not able to associate perioperative metabolite dynamics with clinical outcome measures in an exploratory analysis. As a possible explanation, the effects of IDO-mitigated immunomodulation in cardiac surgery may not necessarily manifest clinically in low-risk patients as investigated in this cohort and depicted in the low mortality rates. Nonetheless, these findings could hold significance in a population with higher risk profiles [47]. Additionally, data from our exploratory analyses suggest that there is a potential association between preoperative serotonin levels and postoperative myocardial infarction. This is interesting because serotonin has been shown to play an important role in platelet aggregation and acute myocardial infarction, and selective serotonin reuptake inhibitors used in the treatment of psychiatric disorders have been proposed as a protective therapy against acute myocardial infarction [52].

Accordingly, our findings lay the foundation for potential future research: As discussed above, an investigation of IDO in combination with T-cell numbers and activation would provide further insight into the mechanistic explanation of our findings and into the dynamics of postoperative immunomodulation. Furthermore, future studies could investigate the role of IDO in high-risk populations, the impact of specific interventions targeting IDO activity, and the exploration of IDO activity in different surgical contexts. Finally, a thorough investigation of preoperative serotonin levels and perioperative myocardial ischemia is warranted, particularly in the context of cardiac surgery. This analysis has several limitations. First, our single-center design restricts the external validity of the findings. Second, the inclusion of only 192 low-risk patients without significant complications may potentially obscure effects. Although we adjusted the paired analysis for a set of confounders, our ability to control for other potential confounders was limited due to the small sample size and low event rates. Third, the immune system is complex, involving various pathways with pro-inflammatory or contra-inflammatory mediators, some of them interacting with each other. Therefore, focusing only on the tryptophan pathway may not adequately capture effects. Fourth, CPB may perioperatively lower the levels of our measured metabolites by dilution through the crystalloid priming volume. Fifth, we used the Kyn/Trp ratio to assess IDO activity. While this is a commonly used method in research, it is influenced by factors such as supplies of nutrients, the presence of multiple cell types, and the interactions of hormones and cytokines [5]. To enhance our results, we additionally measured further metabolites of the pathway. This was made possible by our innovative approach using mass spectrometry. However, the simple quantification of other metabolites by mass spectrometry may not fully reflect their biological activity and functionality. This methodology might also mask proteins that are below their detection limit. In addition, no formal sample size calculation was performed and we therefore consider these results exploratory. On the other hand, this study can inform future sample size calculations. Finally, the cohort studied include predominantly male patients (75.5%), which might skew the results and limit applicability to female patients. Yet, this skewing is in line with the typical patient cohort undergoing cardiac surgery.

## 5. Conclusions

In summary, our findings suggest that patients undergoing cardiopulmonary bypass surgery are likely to exhibit reduced IDO activity. This distinction marks a major difference from pathologies such as cancer or autoimmune disorders in which enhanced IDO activity is well documented. In our analysis, we found no correlation of IDO activity and metabolites with clinical outcome measures. However, further high-quality studies with larger high-risk populations in various surgical settings are warranted to comprehensively evaluate the role of IDO in perioperative immunomodulation in cardiac surgery with CBP, as both perioperative immunosuppression and immune activation are strong drivers of postoperative complications, and thus, it remains possible that IDO might serve as a selective therapeutic target.

## Figures and Tables

**Table 1:** Fragment ions used for quantitation of metabolites.

Name	Fragment Ion (m/z)
Quinolinic acid (QA)	194, 296
Kynurenic acid (KA)	231, 304, 318
Kynurenine (KN)	192, 218, 307, 424
Tryptophan (Trp)	203, 291
Serotonin (5-HT)	174

**Table 2:** Demographic, medical, and surgical study population characteristics.

	All Patients (*n* = 192)	*n*
*Demographics and comorbidities*		
Age (years)	67 [60;73]	192
Sex (Male):	145 (75.5%)	192
BMI (kg/m^2^)	26.1 [23.7;30.4]	192
Diabetes (Yes):	35 (18.2%)	192
Hypertension (Yes):	130 (68.4%)	190
Dyslipidemia (Yes):	111 (58.1%)	191
Nicotine use:		188
Non-smoker	97 (51.6%)	
Smoker/previous smoker	91 (48.4%)	
Adiposities (BMI > 30 kg/m^2^):	52 (27.1%)	192
Preoperative renal disease (Yes):	43 (22.4%)	192
Peripheral vascular disease (Yes):	11 (6.18%)	178
Carotid disease (Yes):	6 (3.7%)	162
Myocardial infarction (Yes):	20 (10.5%)	191
COPD (Yes):	23 (12.1%)	190
NYHA (>I):	131 (68.6%)	191
CCS (>0):	71 (37.6%)	189
Ejection fraction (%)	60 [55;65]	191
EuroSCORE2 (%)	1.7 [0.9;2.9]	184
*Medication*		
Betablockers (Yes):	86 (44.8%)	192
ACE (Yes):	79 (41.1%)	192
ARB (Yes):	47 (24.5%)	192
Aspirin (Yes):	92 (47.9%)	192
Statin (Yes):	105 (54.7%)	192
Steroids (Yes):	7 (3.7%)	192
*Surgery*		
ECC or MiECC:		192
ECC	149 (77.6%)	
MiECC	43 (22.4%)	
Bypass time (min)	104 [80;132]	192
Aortic cross-clamping (min)	69 [52;92]	192
Lowest body temperature (deg C)	33.2 [32.1;33.8]	192
Deep hypothermic cardiac arrest (Yes):	19 (9.95%)	191
No	172 (90.1%)	
Yes	19 (10.0%)	
Operation duration (min)	234 [195;276]	192
Aortic valve (Yes):	86 (44.8%)	192
Mitral valve (Yes):	45 (23.4%)	192
Tricuspid valve (Yes):	17 (8.9%)	192
Coronary artery bypass (Yes):	77 (40.1%)	192
Ascending aortic (Yes):	38 (19.8%)	192
Aortic arch (Yes):	11 (5.7%)	192

**Table 3:** Descriptive summary measures of the Kyn/Trp ratio and the 5 metabolites, and an assessment of the perioperative changes.

Time Point	Kynurenic Acid (KA)	Kynurenine (KN)	Quinolinic Acid (QA)	Tryptophan (Trp)	Serotonin (5-HT)	Kynurenine (KN)/Tryptophan (Trp)
*Summary statistics (median, 95% CI of median)*						
Preoperative	14′784 (95% CI: 12′950 to 16′387)	58′874 (95% CI: 52′453 to 67′771)	21′216 (95% CI: 19′611 to 22′631)	2.15 (95% CI: 2.07 to 2.32)	33′532 (95% CI: 29′274 to 40.859)	27′282 (95% CI: 23′553 to 30′667)
Intraoperative	13′381 (95% CI: 11’992 to 14’843)	41’158 (95% CI: 34’954 to 44’779)	18′991 (95% CI: 16′330 to 22′089)	1.91 (95% CI: 1.77 to 1.99)	32′196 (95% CI: 28′355 to 36′139)	20′538 (95% CI: 17′725 to 23′477)
Postoperative	15′061 (95% CI: 13′677 to 16′502)	51′205 (95% CI: 45′541 to 55′490)	21′250 (95% CI: 19′400 to 23′883)	2.14 (95% CI: 2.08 to 2.23)	35′057 (95% CI: 30′544 to 40′079)	22′211 (95% CI: 20′166 to 25′400)
*Perioperative changes (Kendall’s W as effect size and p-value of Friedman Test)*						
Effect size	0.02 (*p* = 0.0118)	0.19 (*p* < 0.001)	0.02 (*p* = 0.0132)	0.08 (*p* < 0.001)	0.07 (*p* < 0.001)	0.07 (*p* < 0.001)
*Paired changes (Median paired difference with 95% CI, Wilcoxon signed-rank test)*						
Intraoperative–Preoperative	−799 (95% CI: −1′999 to 349, *p* = 0.177)	−18′231 (95% CI: −22′657 to −13′779, *p* < 0.001)	−1′018 (95% CI: −2′843 to 807, *p* = 0.267)	−0.38 (95% CI: −0.53 to −0.26, *p* < 0.001)	−3′720 (95% CI: −6′580 to −966, *p* = 0.011)	−5′090 (95% CI: −6′994 to −3′121, *p* = *p* < 0.001)
Postoperative–Preoperative	408 (95% CI: −838 to 1′673, *p* = 0.487)	−5′834 (95% CI: −10′271 to −1′333, *p* = 0.011)	1′336 (95% CI: −353 to 3′073, *p* = 0.123)	−0.10 (95% CI: −0.21 to 0.01, *p* = 0.075)	1′632 (95% CI: −1′004 to 4′403, *p* = 0.219)	−2′298 (95% CI: −4′028 to −596, *p* = 0.009)
Postoperative–Intraoperative	1′489 (95% CI: 605 to 2′382, *p* = 0.001)	11′070 (95% CI: 8′185 to 14′198, *p* < 0.001)	2′730 (95% CI: 1′427 to 4′115, *p* < 0.001)	0.28 (95% CI: 0.19 to 0.37, *p* < 0.001)	5′015 (95% CI: 2′849 to 7′264, *p* < 0.001)	2′268 (95% CI: 1′062 to 3′628, *p* = *p* < 0.001)

**Table 4:** Adjusted regression coefficients (odds ratios in case of a binary outcome) of standardized biomarker values (see Section 2). Age, sex, and BMI were a priori chosen as confounders.

Outcome	Data	Kynurenic Acid (KA)	Kynurenine (KN)	Quinolinic Acid (QA)	Tryptophan (Trp)	Serotonin (5-HT)	Kynurenine (KN)/ Tryptophan (Trp)
Stroke Incidence 12/192 (6.3%)	Preoperative values	1.16 (95% CI: 0.04 to 5.11) *p* = 0.891	1.13 (95% CI: 0.53 to 1.88) *p* = 0.704	0.94 (95% CI: 0.10 to 2.88) *p* = 0.933	0.95 (95% CI: 0.41 to 1.81) *p* = 0.889	0.77 (95% CI: 0.30 to 1.51) *p* = 0.528	1.02 (95% CI: 0.46 to 1.80) *p* = 0.955
	Postoperative–Preoperative	0.47 (95% CI: 0.11 to 3.13) *p* = 0.338	0.89 (95% CI: 0.41 to 1.75) *p* = 0.773	0.91 (95% CI: 0.39 to 2.11 *p* = 0.842	0.73 (95% CI: 0.39 to 1.47) *p* = 0.340	0.72 (95% CI: 0.34 to 1.42) *p* = 0.363	1.19 (95% CI: 0.58 to 2.26) *p* = 0.637
Myocardial infarction Incidence 6/192 (3.1%)	Preoperative values	0.37 (95% CI: *NA* to 6.55) *p* = 0.804	1.70 (95% CI: 0.58 to 3.65) *p* = 0.218	0.91 (95% CI: 0.00 to 3.90) *p* = 0.945	2.14 (95% CI: 0.78 to 5.52) *p* = 0.104	2.48 (95% CI: 1.00 to 6.28) *p* = 0.041	1.19 (95% CI: 0.25 to 3.07) *p* = 0.794
	Postoperative–Preoperative	2.90 (95% CI: 0.05 to 91.34) *p* = 0.584	0.95 (95% CI: 0.21 to 2.71) *p* = 0.937	0.94 (95% CI: 0.14 to 3.59) *p* = 0.941	1.20 (95% CI: 0.38 to 3.41) *p* = 0.755	1.18 (95% CI: 0.36 to 3.78) *p* = 0.778	0.89 (95% CI: 0.24 to 2.44) *p* = 0.842
30d mortality Incidence 2/192 (1.0%)	Preoperative values	0.00 (95% CI: 0.00 to 16.93) *p* = 0.649	0.64 (95% CI: 0.03 to 2.47) *p* = 0.705	0.56 (95% CI: 0.00 to 8.26) *p* = 0.852	1.32 (95% CI: 0.24 to 4.15) *p* = 0.673	0.65 (95% CI: 0.02 to 2.53) *p* = 0.725	0.45 (95% CI: 0.01 to 2.29) *p* = 0.534
	Postoperative–Preoperative	1.02 (95% CI: 0.06 to 88.85) *p* = 0.993	0.74 (95% CI: 0.16 to 2.90) *p* = 0.727	1.12 (95% CI: 0.19 to 7.02) *p* = 0.916	0.82 (95% CI: 0.24 to 4.32) *p* = 0.782	0.62 (95% CI: 0.11 to 2.70) *p* = 0.572	0.88 (95% CI: 0.25 to 3.64) *p* = 0.871
1-year mortality Incidence 5/192 (2.6%)	Preoperative values	0.00 (95% CI: 0.00 to 2.05) *p* = 0.521	0.57 (95% CI: 0.14 to 1.41) *p* = 0.362	0.19 (95% CI: 0.00 to 2.18) *p* = 0.483	1.32 (95% CI: 0.62 to 2.38) *p* = 0.397	1.02 (95% CI: 0.41 to 1.86) *p* = 0.958	0.29 (95% CI: 0.03 to 1.26) *p* = 0.203
	Postoperative–Preoperative	1.24 (95% CI: 0.18 to 13.30) *p* = 0.867	1.01 (95% CI: 0.44 to 1.82) *p* = 0.972	1.10 (95% CI: 0.42 to 2.55) *p* = 0.838	0.68 (95% CI: 0.35 to 1.45) *p* = 0.283	0.92 (95% CI: 0.41 to 1.80) *p* = 0.839	1.30 (95% CI: 0.54 to 2.67) *p* = 0.528
Length of hospital stay Median 7 days (IQR: 6 to 9 days)	Preoperative values	−0.04 (95% CI: −0.12 to 0.05) *p* = 0.384	0.00 (95% CI: −0.02 to 0.03) *p* = 0.956	−0.03 (95% CI: −0.09 to 0.02) *p* = 0.209	0.00 (95% CI: −0.03 to 0.02) *p* = 0.700	−0.01 (95% CI: −0.04 to 0.01) *p* = 0.347	0.01 (95% CI: −0.02 to 0.03) *p* = 0.641
	Postoperative–Preoperative	0.03 (95% CI: −0.05 to 0.11) *p* = 0.503	0.01 (95% CI: −0.02 to 0.03) *p* = 0.463	0.02 (95% CI: −0.02 to 0.05) *p* = 0.317	−0.01 (95% CI: −0.03 to 0.02) *p* = 0.602	0.01 (95% CI: −0.02 to 0.03) *p* = 0.467	0.01 (95% CI: −0.01 to 0.04) *p* = 0.310

## Data Availability

All data are available from the authors upon reasonable request.

## Supplementary Materials

The following supporting information can be downloaded at: https://www.mdpi.com/article/10.3390/metabo14060334/s1, Figure S1: Graphical summary measures of the Kyn/Trp ratio and the 5 metabolites, and an assessment of the perioperative changes stratified according to CCS. Figure S2: Graphical summary measures of the Kyn/Trp ratio and the 5 metabolites, and an assessment of the perioperative changes stratified according to prior myocardial infarction. Table S1: Adjusted regression coefficients (odds ratios in case of a binary outcome) of standardized biomarker values (see Section 2). Age, sex, BMI, and type of ECC (ECC vs. MiECC). Table S2: Adjusted regression coefficients (odds ratios in case of a binary outcome) of standardized biomarker values (see Section 2). Age, sex, BMI, and aortic valve (No vs. Yes). Table S3: Adjusted regression coefficients (odds ratios in case of a binary outcome) of standardized biomarker values (see Section 2). Age, sex, BMI, and bypass time (in minutes). Table S4: Adjusted regression coefficients (odds ratios in case of a binary outcome) of standardized biomarker values (see Section 2). Age, sex, BMI, and betablockers (No vs. Yes).

## Abbreviations

5-HT	Serotonin
ACE	Angiotensin-converting enzyme inhibitors
AKI	Acute kidney failure
ARB	Angiotensin II receptor blocker
AVR	Aortic valve repair
BMI	Body mass index
CABG	Coronary artery bypass grafting
CCS	Canadian Cardiovascular Society grading
COPD	Chronic obstructive pulmonary disease
CPB	Cardiopulmonary bypass
ECC	Extracorporeal circulation
IDO	Indoleamine 2,3-deoxygenase
Ka	Kynurenic acid
Kyn/Trp	Kynurenine/tryptophan ratio
Kyn	Kynurenine
MiECC	Minimal invasive extracorporeal circulation
MVR	Mitral valve repair
NYHA	New York Heart Association Classification
Pa	Picolinic acid
Qa	Quinolinic acid
SVHD	Singl- ventricle heart disease
TH	T helper
Trp	Tryptophan
TVR	Tricuspid valve repair
