# Supplementary material for: Association of Indoleamine 2,3-Dioxygenase (IDO) Activity with Outcome after Cardiac Surgery in Adult Patients
Source: Metabolites. 2024 Jun 14;14(6):334. doi: 10.3390/metabo14060334 (PMC11205801; doi:10.3390/metabo14060334)
Supplement: Supplementary file 1 [file metabolites-14-00334-s001.zip › metabolites-3049180-supplementary.pdf]

Supplemental figures

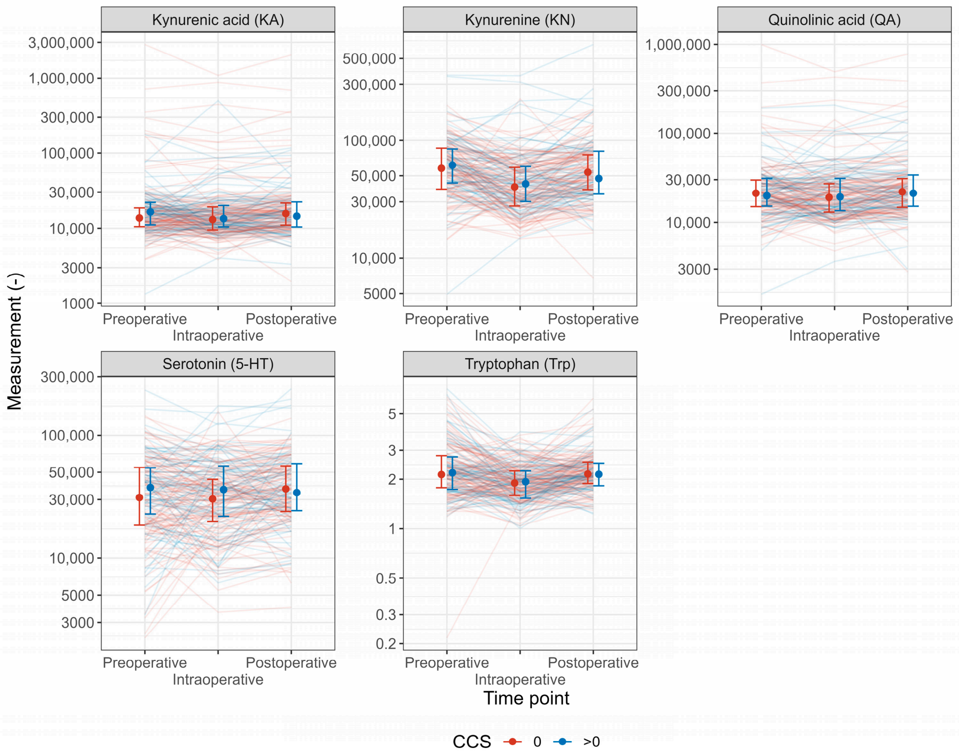

Supplemental Figure S1. Graphical summary measures of the 5 metabolites and assessment of perioperative changes in patients with coronary artery disease.

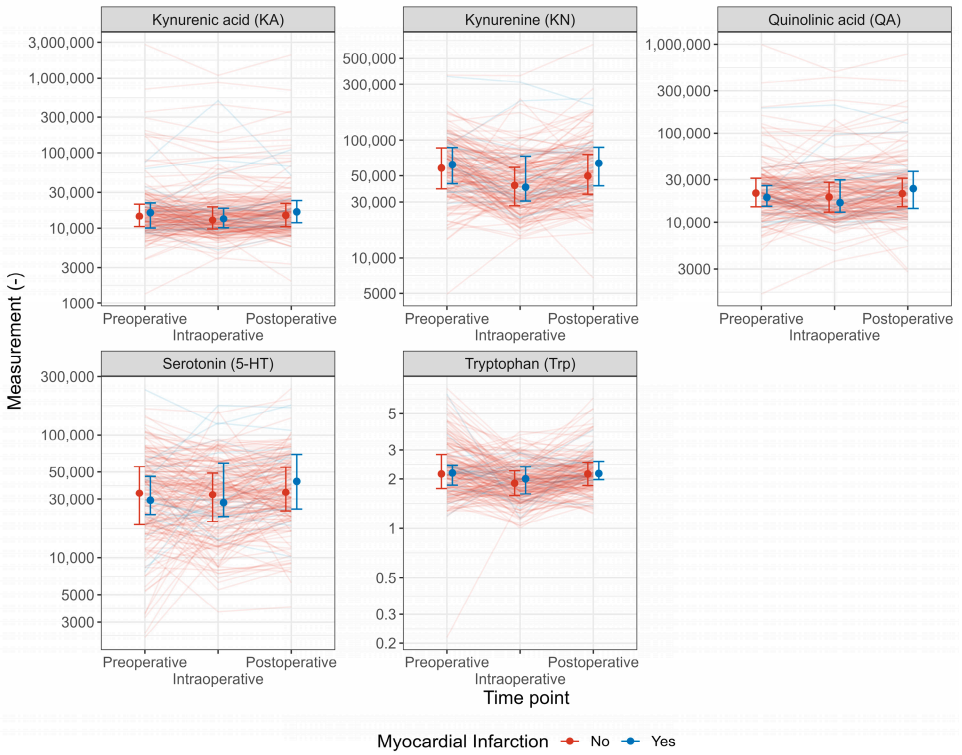

Supplemental Figure S2. Graphical summary measures of the 5 metabolites and assessment of perioperative changes in patients with myocardial infraction.

Supplemental tables

Outcome	Data	Kynurenic acid (KA)†	Kynurenine (KN)	Quinolinic acid (QA) †	Tryptophan (Trp)	Serotonin (5-HT)	Kynurenine (KN)/ Tryptophan (Trp)
**Stroke**<br>Incidence 12/192 (6.3%)	Preoperative values	1.14 (95%-CI: 0.05 to 4.91, p=0.902)	1.14 (95%-CI: 0.54 to 1.88, p=0.665)	0.95 (95%-CI: 0.11 to 2.83, p=0.945)	0.93 (95%-CI: 0.41 to 1.77, p=0.851)	0.78 (95%-CI: 0.31 to 1.50, p=0.535)	1.04 (95%-CI: 0.47 to 1.80, p=0.918)
	Postoperative – Preoperative	0.49 (95%-CI: 0.12 to 3.30, p=0.361)	0.89 (95%-CI: 0.42 to 1.74, p=0.773)	0.94 (95%-CI: 0.40 to 2.19, p=0.886)	0.74 (95%-CI: 0.40 to 1.48, p=0.356)	0.73 (95%-CI: 0.35 to 1.48, p=0.397)	1.20 (95%-CI: 0.59 to 2.29, p=0.617)
**Myocardial infarction**<br>Incidence 6/192 (3.1%)	Preoperative values	0.35 (95%-CI: NA to 5.89, p=0.780)	1.67 (95%-CI: 0.58 to 3.52, p=0.210)	0.84 (95%-CI: 0.00 to 3.67, p=0.906)	2.10 (95%-CI: 0.77 to 5.56, p=0.115)	2.30 (95%-CI: 0.93 to 5.78 p=0.059)	1.23 (95%-CI: 0.24 to 3.04, p=0.738)
	Postoperative – Preoperative	2.27 (95%-CI: 0.05 to 93.17, p=0.678)	0.96 (95%-CI: 0.22 to 2.67, p=0.955)	0.91 (95%-CI: 0.14 to 3.43, p=0.904)	1.08 (95%-CI: 0.35 to 3.09, p=0.890)	1.20 (95%-CI: 0.37 to 4.30, p=0.762)	0.91 (95%-CI: 0.27 to 2.42, p=0.864)
**30d mortality**<br>Incidence 2/192 (1.0%)	Preoperative values	0.00 (95%-CI: 0.00 to 21.71, p=0.689)	0.72 (95%-CI: 0.03 to 2.40, p=0.770)	0.73 (95%-CI: 0.00 to 7.82, p=0.907)	1.21 (95%-CI: 0.22 to 3.85, p=0.768)	0.69 (95%-CI: 0.02 to 2.44, p=0.750)	0.52 (95%-CI: 0.01 to 2.30 p=0.601)
	Postoperative – Preoperative	1.29 (95%-CI: 0.07 to 2398.02, p=0.933)	0.78 (95%-CI: 0.17 to 2.95, p=0.770)	1.34 (95%-CI: 0.21 to 11.93, p=0.805)	0.89 (95%-CI: 0.26 to 5.09, p=0.874)	0.67 (95%-CI: 0.12 to 3.86, p=0.646)	0.92 (95%-CI: 0.26 to 4.31, p=0.918)
**1-year mortality**<br>Incidence 5/192 (2.6%)	Preoperative values	0.01 (95%-CI: 0.00 to 5.36, p=0.647)	0.63 (95%-CI: 0.12 to 1.62, p=0.500)	0.31 (95%-CI: 0.00 to 2.59, p=0.606)	1.71 (95%-CI: 0.77 to 3.45, p=0.139)	1.12 (95%-CI: 0.40 to 2.15, p=0.791)	0.28 (95%-CI: 0.03 to 1.26, p=0.200)
	Postoperative – Preoperative	1.14 (95%-CI: 0.14 to 18.52 p=0.931)	1.00 (95%-CI: 0.36 to 1.99 p=0.994)	1.14 (95%-CI: 0.36 to 3.00 p=0.818)	0.50 (95%-CI: 0.23 to 1.17 p=0.084)	0.78 (95%-CI: 0.29 to 1.90 p=0.622)	1.30 (95%-CI: 0.54 to 2.67 p=0.529)
**Length of hospital stay**<br>Median 7 days (IQR: 6 to 9 days)	Preoperative values	-0.04 (95%-CI: -0.12 to 0.04, p=0.370)	0.00 (95%-CI: -0.02 to 0.03, p=0.892)	-0.04 (95%-CI: -0.09 to 0.02, p=0.196)	-0.01 (95%-CI: -0.03 to 0.02, p=0.553)	-0.01 (95%-CI: -0.04 to 0.01, p=0.366)	0.01 (95%-CI: -0.02 to 0.03, p=0.577)
	Postoperative – Preoperative	0.03 (95%-CI: -0.05 to 0.12, p=0.417)	0.01 (95%-CI: -0.02 to 0.03, p=0.466)	0.02 (95%-CI: -0.01 to 0.06, p=0.201)	0.00 (95%-CI: -0.03 to 0.02, p=0.716)	0.01 (95%-CI: -0.01 to 0.04, p=0.280)	0.01 (95%-CI: -0.01 to 0.04, p=0.312)

Supplemental Table S1. Adjusted regression coefficients (odds ratios in case of a binary outcome) of standardized biomarker values (see methods). Age, sex, BMI and type of ECC (ECC vs. MiECC).

Outcome	Data	Kynurenic acid (KA)†	Kynurenine (KN)	Quinolinic acid (QA) †	Tryptophan (Trp)	Serotonin (5-HT)	Kynurenine (KN)/ Tryptophan (Trp)
**Stroke** Incidence 12/192 (6.3%)	Preoperative values	1.22 (95%-CI: 0.04 to 5.63,p=0.856)	1.12 (95%-CI: 0.51 to 1.87,p=0.734)	0.95 (95%-CI: 0.09 to 3.06,p=0.946)	0.95 (95%-CI: 0.41 to 1.80,p=0.884)	0.77 (95%-CI: 0.30 to 1.51,p=0.526)	1.00 (95%-CI: 0.45 to 1.79,p=0.991)
	Postoperative – Preoperative	0.46 (95%-CI: 0.11 to 3.13,p=0.331)	0.88 (95%-CI: 0.41 to 1.73,p=0.752)	0.91 (95%-CI: 0.38 to 2.11,p=0.842)	0.73 (95%-CI: 0.39 to 1.46,p=0.335)	0.72 (95%-CI: 0.35 to 1.42,p=0.363)	1.19 (95%-CI: 0.57 to 2.26,p=0.638)
**Myocardial infarction** Incidence 6/192 (3.1%)	Preoperative values	0.41 (95%-CI: NA to 12.28,p=0.854)	1.51 (95%-CI: 0.47 to 3.43,p=0.390)	1.07 (95%-CI: 0.00 to 6.27,p=0.967)	2.04 (95%-CI: 0.72 to 5.28,p=0.133)	2.34 (95%-CI: 0.89 to 6.49,p=0.083)	1.03 (95%-CI: 0.22 to 2.87,p=0.960)
	Postoperative – Preoperative	6.03 (95%-CI: 0.06 to 247.97,p=0.381)	1.16 (95%-CI: 0.24 to 3.12,p=0.826)	1.12 (95%-CI: 0.16 to 4.63,p=0.897)	1.27 (95%-CI: 0.42 to 3.47,p=0.658)	1.49 (95%-CI: 0.44 to 4.86,p=0.511)	1.05 (95%-CI: 0.23 to 3.34,p=0.945)
**30d mortality** Incidence 2/192 (1.0%)	Preoperative values	0.00 (95%-CI: 0.00 to 5.51,p=0.613)	0.60 (95%-CI: 0.02 to 2.53,p=0.681)	0.49 (95%-CI: 0.00 to 9.75,p=0.838)	1.34 (95%-CI: 0.24 to 4.14,p=0.651)	0.65 (95%-CI: 0.02 to 2.71,p=0.727)	0.41 (95%-CI: 0.01 to 2.23,p=0.501)
	Postoperative – Preoperative	0.98 (95%-CI: 0.05 to 87.98,p=0.993)	0.71 (95%-CI: 0.12 to 2.89,p=0.701)	1.08 (95%-CI: 0.16 to 7.00,p=0.942)	0.81 (95%-CI: 0.24 to 4.40,p=0.763)	0.61 (95%-CI: 0.11 to 2.69,p=0.562)	0.87 (95%-CI: 0.21 to 3.62,p=0.862)
**1-year mortality** Incidence 5/192 (2.6%)	Preoperative values	0.01 (95%-CI: 0.00 to 5.90,p=0.637)	0.62 (95%-CI: 0.12 to 1.61,p=0.486)	0.29 (95%-CI: 0.00 to 2.74,p=0.600)	1.69 (95%-CI: 0.77 to 3.32,p=0.140)	1.11 (95%-CI: 0.40 to 2.15,p=0.800)	0.28 (95%-CI: 0.03 to 1.25,p=0.199)
	Postoperative – Preoperative	1.13 (95%-CI: 0.13 to 16.73,p=0.939)	1.00 (95%-CI: 0.36 to 1.99,p=0.997)	1.13 (95%-CI: 0.36 to 2.94,p=0.831)	0.51 (95%-CI: 0.24 to 1.17,p=0.085)	0.78 (95%-CI: 0.29 to 1.83,p=0.621)	1.30 (95%-CI: 0.54 to 2.67,p=0.526)
**Length of hospital stay** Median 7 days (IQR: 6 to 9 days)	Preoperative values	-0.04 (95%-CI: -0.12 to 0.05,p=0.373)	0.00 (95%-CI: -0.02 to 0.03,p=0.948)	-0.04 (95%-CI: -0.09 to 0.02,p=0.202)	-0.01 (95%-CI: -0.03 to 0.02,p=0.697)	-0.01 (95%-CI: -0.04 to 0.01,p=0.353)	0.01 (95%-CI: -0.02 to 0.03,p=0.633)
	Postoperative – Preoperative	0.03 (95%-CI: -0.05 to 0.11,p=0.497)	0.01 (95%-CI: -0.02 to 0.03,p=0.456)	0.02 (95%-CI: -0.02 to 0.05,p=0.308)	-0.01 (95%-CI: -0.03 to 0.02,p=0.621)	0.01 (95%-CI: -0.02 to 0.03,p=0.463)	0.01 (95%-CI: -0.01 to 0.04,p=0.314)

Supplemental Table S2. Adjusted regression coefficients (odds ratios in case of a binary outcome) of standardized biomarker values (see methods). Age, sex, BMI and aortic valve (No vs. Yes).

Outcome	Data	Kynurenic acid (KA)†	Kynurenine (KN)	Quinolinic acid (QA) †	Tryptophan (Trp)	Serotonin (5-HT)	Kynurenine (KN)/ Tryptophan (Trp)
**Stroke** Incidence 12/192 (6.3%)	Preoperative values	1.16 (95%-CI: 0.04 to 5.47,p=0.896)	1.17 (95%-CI: 0.57 to 1.93,p=0.605)	0.95 (95%-CI: 0.10 to 3.00,p=0.951)	0.81 (95%-CI: 0.33 to 1.58,p=0.582)	0.83 (95%-CI: 0.34 to 1.53,p=0.629)	1.12 (95%-CI: 0.51 to 2.00,p=0.751)
	Postoperative – Preoperative	0.49 (95%-CI: 0.11 to 3.66,p=0.418)	0.93 (95%-CI: 0.43 to 1.81,p=0.841)	0.92 (95%-CI: 0.39 to 2.10,p=0.847)	0.85 (95%-CI: 0.44 to 1.81,p=0.642)	0.75 (95%-CI: 0.36 to 1.50,p=0.431)	1.16 (95%-CI: 0.54 to 2.25,p=0.685)
**Myocardial infarction** Incidence 6/192 (3.1%)	Preoperative values	0.19 (95%-CI: NA to 7.01,p=0.727)	1.87 (95%-CI: 0.62 to 4.36,p=0.168)	0.67 (95%-CI: 0.00 to 4.26,p=0.824)	1.95 (95%-CI: 0.74 to 4.95,p=0.145)	2.33 (95%-CI: 0.96 to 5.85,p=0.051)	1.34 (95%-CI: 0.25 to 3.83,p=0.673)
	Postoperative – Preoperative	2.19 (95%-CI: 0.04 to 74.16,p=0.671)	0.99 (95%-CI: 0.21 to 3.10,p=0.991)	1.01 (95%-CI: 0.17 to 3.59,p=0.984)	1.40 (95%-CI: 0.42 to 4.60,p=0.604)	1.27 (95%-CI: 0.38 to 4.20,p=0.692)	0.87 (95%-CI: 0.21 to 2.47,p=0.823)
**30d mortality** Incidence 2/192 (1.0%)	Preoperative values	0.00 (95%-CI: 0.00 to 11.60,p=0.610)	0.75 (95%-CI: 0.03 to 2.51,p=0.794)	0.51 (95%-CI: 0.00 to 8.35,p=0.833)	1.11 (95%-CI: 0.16 to 4.04,p=0.889)	0.70 (95%-CI: 0.03 to 2.47,p=0.758)	0.54 (95%-CI: 0.01 to 2.54,p=0.628)
	Postoperative – Preoperative	1.58 (95%-CI: 0.05 to 142.25,p=0.881)	0.82 (95%-CI: 0.17 to 3.29,p=0.817)	1.24 (95%-CI: 0.20 to 7.84,p=0.841)	1.04 (95%-CI: 0.25 to 7.10,p=0.967)	0.67 (95%-CI: 0.12 to 3.01,p=0.637)	0.89 (95%-CI: 0.21 to 3.98,p=0.894)
**1-year mortality** Incidence 5/192 (2.6%)	Preoperative values	0.02 (95%-CI: 0.00 to 5.31,p=0.650)	0.62 (95%-CI: 0.12 to 1.63,p=0.493)	0.30 (95%-CI: 0.00 to 2.55,p=0.607)	1.75 (95%-CI: 0.78 to 3.54,p=0.128)	1.12 (95%-CI: 0.39 to 2.22,p=0.798)	0.29 (95%-CI: 0.03 to 1.26,p=0.204)
	Postoperative – Preoperative	1.15 (95%-CI: 0.14 to 17.75,p=0.927)	1.02 (95%-CI: 0.36 to 1.99,p=0.971)	1.17 (95%-CI: 0.37 to 3.09,p=0.789)	0.50 (95%-CI: 0.23 to 1.16,p=0.081)	0.79 (95%-CI: 0.29 to 1.84,p=0.634)	1.32 (95%-CI: 0.56 to 2.72,p=0.502)
**Length of hospital stay** Median 7 days (IQR: 6 to 9 days)	Preoperative values	-0.04 (95%-CI: -0.12 to 0.04,p=0.350)	0.00 (95%-CI: -0.02 to 0.03,p=0.920)	-0.04 (95%-CI: -0.09 to 0.02,p=0.177)	-0.01 (95%-CI: -0.03 to 0.02,p=0.459)	-0.01 (95%-CI: -0.04 to 0.01,p=0.319)	0.01 (95%-CI: -0.02 to 0.03,p=0.502)
	Postoperative – Preoperative	0.03 (95%-CI: -0.05 to 0.11,p=0.473)	0.01 (95%-CI: -0.02 to 0.03,p=0.517)	0.02 (95%-CI: -0.02 to 0.05,p=0.319)	0.00 (95%-CI: -0.03 to 0.02,p=0.855)	0.01 (95%-CI: -0.01 to 0.04,p=0.421)	0.01 (95%-CI: -0.01 to 0.03,p=0.431)

Supplemental Table S3. Adjusted regression coefficients (odds ratios in case of a binary outcome) of standardized biomarker values (see methods). Age, sex, BMI and bypass time (in minutes).

Outcome	Data	Kynurenic acid (KA)†	Kynurenine (KN)	Quinolinic acid (QA) †	Tryptophan (Trp)	Serotonin (5-HT)	Kynurenine (KN)/ Tryptophan (Trp)
**Stroke** Incidence 12/192 (6.3%)	Preoperative values	1.10 (95%-CI: 0.04 to 5.00,p=0.929)	1.12 (95%-CI: 0.53 to 1.87,p=0.723)	0.90 (95%-CI: 0.10 to 2.83,p=0.897)	0.94 (95%-CI: 0.41 to 1.79,p=0.875)	0.77 (95%-CI: 0.30 to 1.49,p=0.523)	1.02 (95%-CI: 0.46 to 1.79,p=0.959)
	Postoperative – Preoperative	0.49 (95%-CI: 0.11 to 3.41,p=0.378)	0.91 (95%-CI: 0.42 to 1.76,p=0.803)	0.95 (95%-CI: 0.40 to 2.23,p=0.906)	0.74 (95%-CI: 0.39 to 1.48,p=0.358)	0.72 (95%-CI: 0.35 to 1.46,p=0.384)	1.20 (95%-CI: 0.59 to 2.29,p=0.613)
**Myocardial infarction** Incidence 6/192 (3.1%)	Preoperative values	0.51 (95%-CI: NA to 6.18,p=0.852)	1.76 (95%-CI: 0.66 to 3.79,p=0.152)	0.98 (95%-CI: NA to 3.80,p=0.987)	1.86 (95%-CI: 0.65 to 4.86,p=0.197)	2.46 (95%-CI: 1.02 to 6.45,p=0.041)	1.40 (95%-CI: 0.33 to 3.23,p=0.511)
	Postoperative – Preoperative	6.02 (95%-CI: 0.07 to 357.83,p=0.414)	0.93 (95%-CI: 0.21 to 2.51,p=0.913)	1.32 (95%-CI: 0.19 to 5.50,p=0.751)	1.26 (95%-CI: 0.44 to 3.21,p=0.642)	1.36 (95%-CI: 0.39 to 4.73,p=0.627)	0.82 (95%-CI: 0.23 to 2.25,p=0.730)
**30d mortality** Incidence 2/192 (1.0%)	Preoperative values	0.00 (95%-CI: 0.00 to 22.33,p=0.692)	0.63 (95%-CI: 0.03 to 2.38,p=0.694)	0.56 (95%-CI: 0.00 to 7.66,p=0.842)	1.29 (95%-CI: 0.23 to 3.98,p=0.689)	0.66 (95%-CI: 0.02 to 2.44,p=0.731)	0.44 (95%-CI: 0.01 to 2.27,p=0.528)
	Postoperative – Preoperative	1.21 (95%-CI: 0.07 to 248.58,p=0.945)	0.80 (95%-CI: 0.17 to 3.03,p=0.784)	1.24 (95%-CI: 0.21 to 10.37,p=0.849)	0.82 (95%-CI: 0.25 to 4.14,p=0.778)	0.64 (95%-CI: 0.12 to 3.01,p=0.600)	0.93 (95%-CI: 0.25 to 4.21,p=0.927)
**1-year mortality** Incidence 5/192 (2.6%)	Preoperative values	0.01 (95%-CI: 0.00 to 5.09,p=0.634)	0.63 (95%-CI: 0.13 to 1.61,p=0.500)	0.29 (95%-CI: 0.00 to 2.55,p=0.594)	1.69 (95%-CI: 0.76 to 3.44,p=0.150)	1.11 (95%-CI: 0.40 to 2.15,p=0.805)	0.29 (95%-CI: 0.03 to 1.27,p=0.208)
	Postoperative – Preoperative	1.22 (95%-CI: 0.14 to 21.01,p=0.898)	1.02 (95%-CI: 0.36 to 1.99,p=0.969)	1.18 (95%-CI: 0.36 to 3.17,p=0.776)	0.51 (95%-CI: 0.22 to 1.18,p=0.093)	0.79 (95%-CI: 0.29 to 1.93,p=0.646)	1.31 (95%-CI: 0.55 to 2.68,p=0.516)
**Length of hospital stay** Median 7 days (IQR: 6 to 9 days)	Preoperative values	-0.03 (95%-CI: -0.11 to 0.05,p=0.488)	0.00 (95%-CI: -0.02 to 0.03,p=0.852)	-0.03 (95%-CI: -0.08 to 0.02,p=0.289)	0.00 (95%-CI: -0.03 to 0.02,p=0.911)	-0.01 (95%-CI: -0.04 to 0.01,p=0.398)	0.01 (95%-CI: -0.02 to 0.03,p=0.652)
	Postoperative – Preoperative	0.01 (95%-CI: -0.07 to 0.10,p=0.724)	0.01 (95%-CI: -0.02 to 0.03,p=0.669)	0.01 (95%-CI: -0.02 to 0.04,p=0.538)	-0.01 (95%-CI: -0.03 to 0.02,p=0.461)	0.00 (95%-CI: -0.02 to 0.03,p=0.732)	0.01 (95%-CI: -0.01 to 0.04,p=0.421)

Supplemental Table S4. Adjusted regression coefficients (odds ratios in case of a binary outcome) of standardized biomarker values (see methods). Age, sex, BMI and betab
